# Case Report: Disseminated *Mycobacterium intracellulare* Infection With More Than 1-Year Follow-Up in a Young Boy With *IFNGR1* Deficiency

**DOI:** 10.3389/fped.2022.761265

**Published:** 2022-02-28

**Authors:** Jihang Jia, Yu Zhu, Qin Guo, Chaomin Wan

**Affiliations:** ^1^Department of Pediatrics, West China Second University Hospital, Sichuan University, Chengdu, China; ^2^Key Laboratory of Birth Defects and Related Diseases of Women and Children, Sichuan University, Ministry of Education, Chengdu, China

**Keywords:** *Mycobacterium intracellulare*, *Mycobacterium avium* complex, non-tuberculous mycobacterium, primary immunodeficiency diseases, interferon-γ receptor 1 deficiency, Langerhans cell histiocytosis (LCH)

## Abstract

All members of the genus *Mycobacterium* are collectively labeled as “non-tuberculous mycobacterium” (NTM), with the exception of the *Mycobacterium tuberculosis* complex and *M. leprae*. Recently, the incidence of NTM infection and number of cases have been increasing, but their identification remains difficult in some countries. Usually, NTM infections and diseases are associated with primary immunodeficiency diseases (PIDs), and their prognoses can be improved with a timely diagnosis and appropriate treatment. Here, we report a case of a 3-year-old boy with disseminated NTM disease (*Mycobacterium intracellulare*) and interferon-γ receptor 1 (*IFNGR1*) deficiency. He presented with skin and soft-tissue disease, disseminated osteomyelitis, and pulmonary disease. Initially, we suspected an infection due to the Bacillus Calmette–Guérin vaccine but later suspected Langerhans cell histiocytosis. Following oral treatment of azithromycin, rifampicin, and ethambutol, his condition improved progressively according to clinical and imaging manifestations. This case highlights the importance of early identification of the pathogen in a timely prescription of specific treatments in PIDs patients. We also discuss our experience of treatment of *M. intracellulare* disease in patients with *IFNGR1* deficiency.

## Introduction

In recent years, the incidence of non-tuberculous mycobacterium (NTM) infections in children and adolescents has been increasing. NTM infections have four distinct clinical syndromes: skin and soft-tissue disease, lymphadenitis, disseminated disease, and pulmonary disease (1). In children, lymphadenitis is its most common form (1); disseminated disease is uncommon and indicates primary immunodeficiency diseases (PIDs).

PIDs are a group of clinical syndromes in which genetic mutations cause defects in immune organs, immune cells, and immunologically active molecules, thereby resulting in abnormal immune function. Several types of PIDs are susceptible to mycobacteria infections: chronic granulomatous disease (CGD), severe combined immunodeficiency disease (SCID), Mendelian susceptibility to mycobacterial disease (MSMD), and hyper-immunoglobulin (Ig)M syndrome.

MSMD is caused by a single gene mutation and is highly and selectively susceptible to weakly virulent mycobacteria, such as NTM and Bacillus Calmette–Guérin (BCG) vaccines (2). Affected patients display defects in initiation of interferon (IFN)-γ-based cellular responses (2). Since 1996, the genetic etiology of MSMD has been linked to mutations in 15 genes (13 autosomal genes and two X-linked genes). One such mutation is IFN-γ receptor 1 (*IFNGR1*) deficiency (2). Overall, disseminated NTM diseases underlying MSMD are a challenge in previously healthy children because of their non-specific symptoms, and optimal management requires a more accurate definition (1).

Here, we report a young patient with disseminated NTM disease (*Mycobacterium intracellulare* infection) belonging to the *Mycobacterium avium* complex (MAC). This patient had a hereditary deficiency of *IFNGR1*, and he presented with skin and soft-tissue disease, disseminated osteomyelitis, and pulmonary disease. Bacillus Calmette–Guérin infection (BCG-itis) and Langerhans cell histiocytosis (LCH) were considered as differential diagnoses. We also discuss treatment for disseminated MAC disease associated with the *IFNGR1* deficiency to better understand its diagnosis and management in children with underlying immunodeficiencies.

## Case Description

A boy aged 3 years and 7 months presented with lumps that had persisted for >7 months and intermittent fever. His family history did not contain recurrent infections or immunodeficiencies (e.g., lymphoproliferative or autoimmune diseases). The patient and his family had no psychosocial history. He had a healthy 10-year-old brother. He was immunized with the BCG vaccine at birth (October 2016). Upon vaccination, he presented a subcutaneous abscess along with a purulent lump (4 × 5 cm) in the left axilla, which was accompanied with local redness and tenderness. A biopsy of the lump indicated tuberculosis, and BCG-itis was suspected, but treatment was not given. At 3 months of age, computed tomography (CT) suggested tuberculosis in axillary lymph nodes (Supplementary Figures 1–6). Isoniazid was used topically for 4 months over the local lump until it reduced in size to 1 × 2 cm.

**Figure 1:**
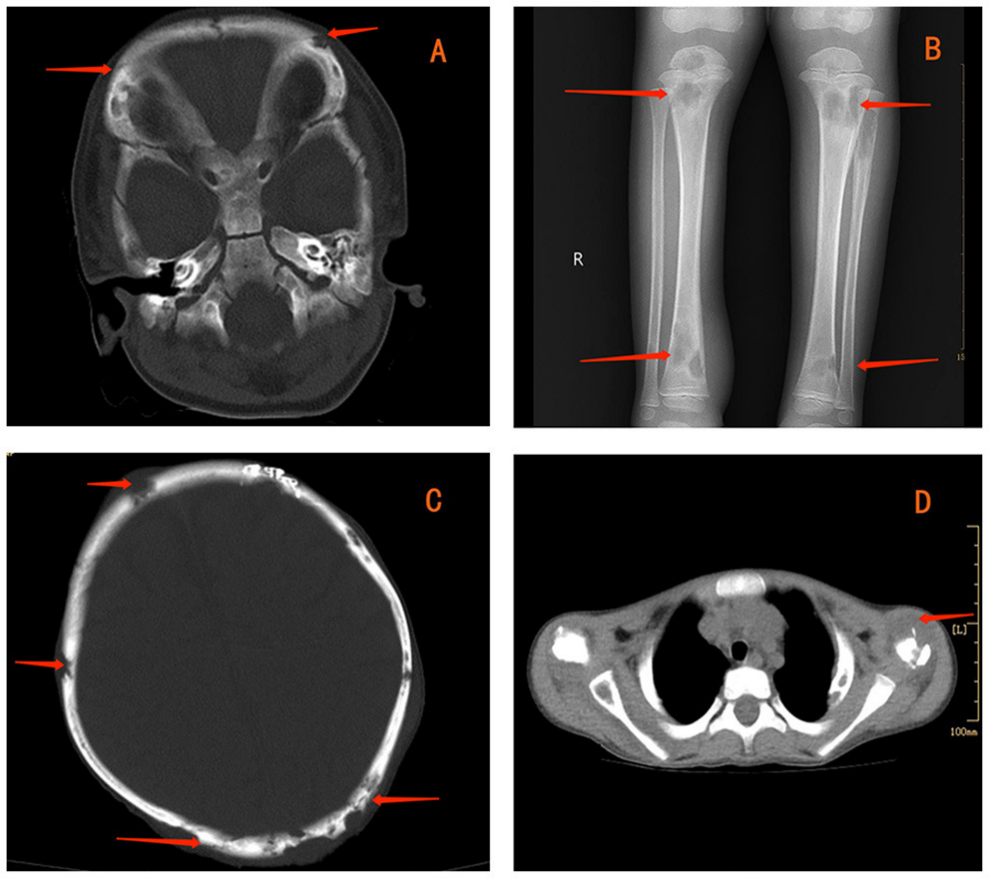
Contrast**-**enhanced computed tomography (CT) showing multiple lytic bone lesions in the skull **(A)**. Radiography reveals multiple lytic bone lesions in bilateral tibias **(B)**. CT indicates osteolytic lesions in the skull **(C)** and left clavicle **(D)**.

At 7 months of age, the boy was re-admitted to hospital because of the left-axillary lump as well as a pale complexion of 1-month duration. He had high-grade fever, anemia, eczematous rashes, and hepatosplenomegaly without lesions upon hospitalization. A sputum smear for acid-fast bacillus (AFB) and an IFN-γ release assay (IGRA), the TSPOT.TB test, were negative. The Mantoux test (purified protein derivative [PPD]) was positive, whereas the blood culture was negative. Subsequently, the child underwent a biopsy in the left chest wall. The biopsy smear for AFB was positive, but the quantitative polymerase chain reaction (qPCR) for *Mycobacterium tuberculosis* (MTB) was negative. A bone-marrow puncture failed twice. Contrast-enhanced CT revealed lymph nodes on both sides of the axilla to be increasing progressively in size, number, and necrosis; he also had multiple lytic bony lesions in his skull (Figure 1A). Imaging strongly implied a diagnosis of LCH. The immunohistochemical staining of skin-biopsy specimens was negative for cluster of differentiation (CD)20, S-100, CD1a, CD207 (langerin), and CD68, but positive for CD3ε. Hence, a diagnosis of LCH was not made, and a tentative diagnosis of lymph-node tuberculosis was considered. He would receive chemotherapy once a definitive diagnosis of LCH was made, so we decided to give him prophylactic anti-tuberculosis therapy in advance. Etiological evidence for lymph-node tuberculosis was not detected and, due to his young age, ethambutol was not administered to avoid retrobulbar optic neuritis and other adverse effects. Then, he received triple antimycobacterial therapy (rifampicin, isoniazid, and pyrazinamide). However, after 3 months, his parents stopped the drug treatment without our permission and apparent improvement.

We suggested an additional bone-marrow puncture and genetic screening for juvenile myelomonocytic leukemia. However, his parents refused further examination, and he was discharged from hospital. For the next 2 years, the child remained asymptomatic without deterioration of bony lesions, but repeated infections of the respiratory tract were observed.

**Figure 2:**
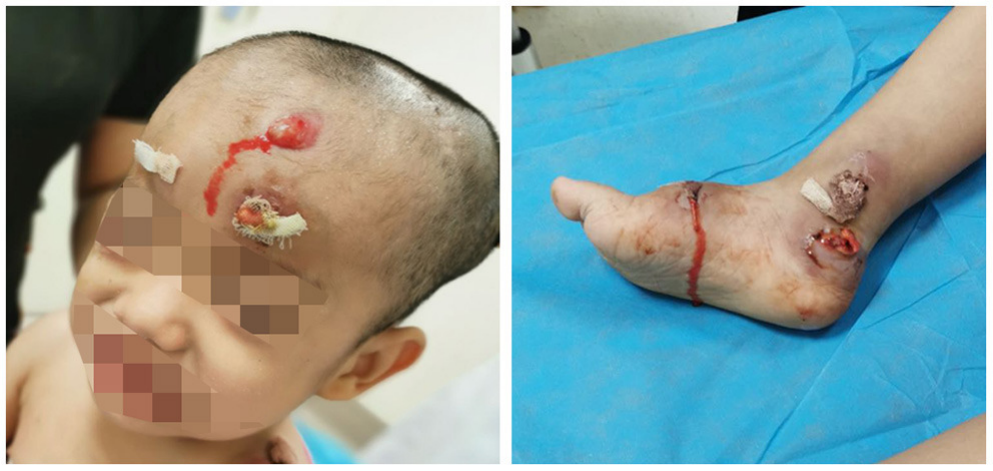
Lumps and purulent secreta due to *Mycobacterium intracellulare* infection.

At approximately 3 years of age, he developed a painless swelling over the occipital bone. Initially, fever or headache were not observed. Contrast-enhanced CT and radiography again revealed multiple lytic bony lesions throughout the body. Then, the patient began to present limited motion of the upper and lower limbs that was accompanied with pain. He underwent two surgical interventions to treat the swelling and two scalp biopsies at other hospitals, but he experienced only transient improvement. The histopathology of the scalp biopsy revealed hyperplastic fibrous tissue surrounded by histiocytes, neutrophils, plasmocytes, lymphocytes, and eosinophils. The immunohistochemical results were negative for S-100, CD1a, and CD207 (langerin), but positive for CD20, CD3ε, and CD68. A genetic test for BRAF^V600E^ was negative. A diagnosis of LCH could not be made without definitive evidence. Within the next 2 months, the lumps increased progressively in size and number with pain in the skull, left scapular region, and limbs after surgical interventions. Moderate- or low-grade intermittent fevers occurred irregularly as the disease progressed. Furthermore, the lumps erupted spontaneously, and purulent secreta became visible (Figure 2).

In mid-May 2020, the child was admitted to our hospital because the systemic lumps were deteriorating. Physical examination revealed numerous subcutaneous lumps throughout his body; they were soft-to-hard in consistency, with the largest being 4 × 3 cm in size. Multiple nodular swellings were observed in the neck and groin. He had limited motion of the legs and right arm, and he could not walk. A slight weight loss of 1 kg was reported.

The laboratory work-up reported levels of leukocytes (35.2 × 10^9^/L), thrombocytes (624 × 10^9^/L), C-reactive protein (223.9 mg/L), erythrocyte sedimentation rate (120 mm/h), and hemoglobin (72 g/L). Upon hospital admission, he was treated with ceftriaxone because of high infectious indexes. Values for humoral and cellular immune parameters were within the normal range for his age. PPD remained positive. The blood test for IGRAs was negative, as were bacterial and fungal cultures of blood. Radiography revealed multiple lytic bone lesions in the bilateral tibias (Figure 1B), femurs, fibulas, and right calcaneus. Contrast-enhanced CT revealed osteolytic lesions in the skull (Figure 1C), right scapula, bilateral humeri, left clavicle (Figure 1D), ribs on either side of the chest, and thoracic vertebral bodies. Streak niduses were observed in both lungs (Supplementary Figures 7–9). These results (and positron emission tomography–computed tomography) suggested a high probability of LCH. Vancomycin was added later to therapy because the child developed a high fever, but his fever was uncontrolled.

The patient underwent surgical drainage of the lumps and four biopsies for local treatment. The histopathology of the pus and bone biopsies indicated granuloma without neoplastic lesions or infiltration with inflammatory cells, which did not conform to the pathological characteristics of LCH. However, intraoperative findings confirmed the diagnosis of osteomyelitis. This development prompted us to reconsider an infectious disease as the most likely cause of the osteomyelitis. Secreta smears for AFB and fungi were negative. Culturing of secreta indicated methicillin-resistant *Staphylococcus aureus*. A bone-marrow puncture was conducted again, and the culture indicated methicillin-resistant coagulase-negative staphylococci. Despite continued anti-infection treatments combined with meropenem, his high fever did not improve. The diagnosis became more perplexing.

**Figure 3:**
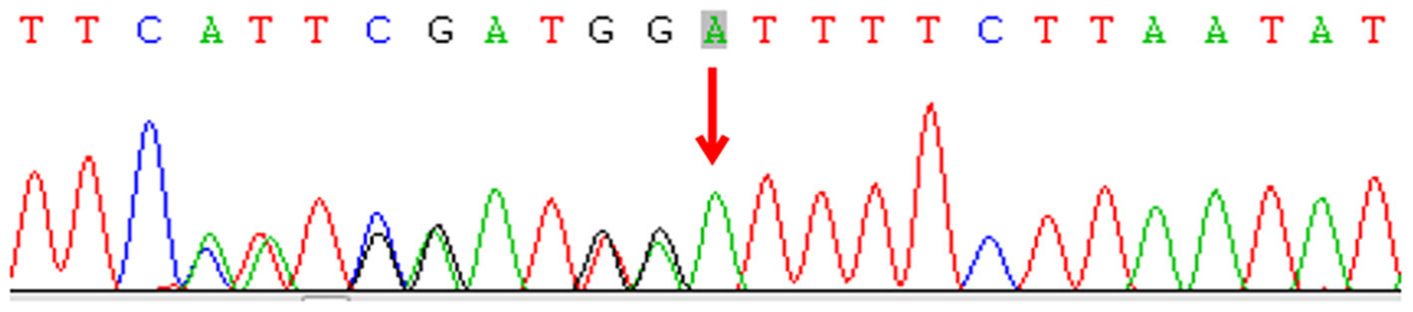
Sequencing of the interferon-gamma receptor-1 gene of the patient.

Subsequently, we undertook metagenomic next-generation sequencing (mNGS) on the drainage pus using the Ion Torrent S5 sequencing platform (Thermo Fisher, Waltham, MA, USA), which defined *M. intracellulare* (one of the members of the MAC) as the causative pathogen. Culturing of the pus specimen revealed atypical mycobacteria identified as a MAC species, and a qPCR for *M. intracellulare* verified the results. A final diagnosis of disseminated NTM infection was made, and the child was started on rifampicin, ethambutol, and azithromycin. He responded well without apparent adverse effects. His disseminated MAC infection led to the hypothesis of a primary immunodeficiency. Subsequent genetic sequencing revealed a heterozygous deletion mutation c.819-822 del4 (p. N274Hfs^*^2) in exon 6 of *IFNGR1* (Figure 3). Unfortunately, the plasma level of IFN-γ was not measured, and his parents did not receive genetic screening.

**Figure 4:**
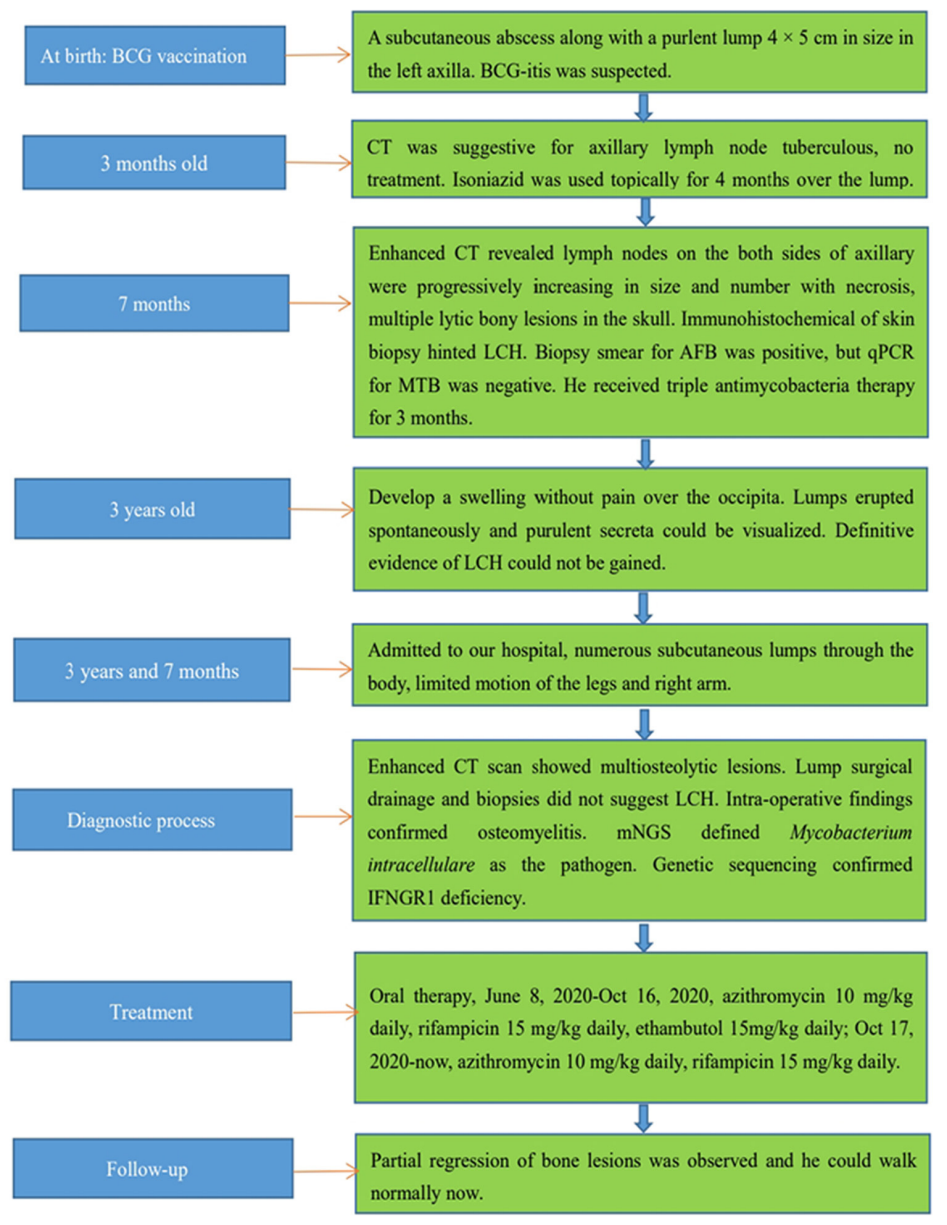
Clinical course of the patient.

Daily oral treatment with azithromycin (10 mg/kg), rifampicin (15 mg/kg), and ethambutol (15 mg/kg) was initiated on 8 June 2020 in the absence of susceptibility tests for *M. intracellulare*. The child did not exhibit adverse reactions. One month later, the culture for *M. intracellulare* was positive, but the drug-sensitivity test indicated azithromycin resistance. However, his condition improved progressively with resolution of the lumps, and therapy was not modified. Four months later, the culture and PCR for mycobacteria were negative, and contrast-enhanced CT showed improvement of bony lesions. Consequently, ethambutol was stopped. Azithromycin and rifampicin were maintained without adverse reactions. The child was monitored routinely for osteomyelitis and liver function in outpatient follow-ups using CT. Partial regression of bone lesions was observed in the most recent CT images, and the child could walk normally. Currently, the patient is taking azithromycin and rifampicin *via* the oral route. In the future, radiography, CT, and liver-function tests will be carried out routinely to monitor his progress. Moreover, culture, qPCR, and other laboratory tests related to mycobacteria will be conducted routinely, and abnormal lumps or bone motion will be monitored. In addition, electrocardiography will be checked for a possible prolonged QT interval due to azithromycin treatment. Figure 4 illustrates the clinical course of this child.

## Discussion

The BCG vaccine induces an immune response that provides protection against more virulent MTB. For most children, the BCG vaccine is safe, but complications have arisen following the vaccination of infants with PIDs (3). In China, localized BCG-itis has been observed in about 10.68/100,000 people, and disseminated infections observed in about 0.21/100,000 (4). Disseminated BCG infection should be considered as evidence for an acquired immunodeficiency disease or PID, and it is associated with a high prevalence of mortality (3). However, microbiological evidence is not always available, and the diagnosis is often based solely on clinical findings. Moreover, some PIDs (e.g., SCID, CGD, and MSMD) cannot be detected and/or confirmed until BCG-related adverse effects emerge. In our patient, BCG infection was suspected initially, but microbiological evidence was not obtained. In addition, empirical anti-tuberculosis therapy was inappropriate because BCG is naturally resistant to pyrazinamide. PIDs were not explored further due to the later suspicion of LCH.

LCH (also known as “histiocytosis X”) is characterized by clonal proliferation and abnormal accumulation of immature histiocytes in various tissues and organs, along with an associated inflammatory infiltrate (5). LCH lesions have well-defined histological characteristics in sections stained with hematoxylin and eosin, but positive CD1a and/or CD207 (langerin) staining of lesion cells is required for a definitive diagnosis (6). Recent progress in the biological understanding of the disease supports the classification of LCH as an inflammatory myeloid neoplasia (7). LCH pathogenic cells are defined by universal activation of the mitogen-activated protein kinase (MAPK) signaling pathway. The most common alteration in LCH is a somatic BRAF^V600E^ mutation, which is present in approximately two-thirds of cases, followed by MAP2K1 mutations (8). The clinical presentation of LCH is widely variable, ranging from single indolent lesions to an explosive multisystem disease. The skeleton is involved most frequently, accounting for 80% of cases (8). The most common site involving the bones is the skull (which is affected in more than two-thirds of patients with bone disease) followed by the spine, limbs, and pelvis (9). Bone scintigraphy reveals lytic lesions without marginal sclerosis, with or without a periosteal reaction.

Our patient did not demonstrate the typical immunohistological characteristics of LCH because he had negative immunostaining of CD1a and CD207 (langerin), and his genetic test for BRAF^V600E^ was also negative. LCH was highly suspected due to radiological features, but a final diagnosis was not made, and chemotherapy was not started. Notably, MAC-induced multiple osteomyelitis closely mimicked LCH in the images and clinical manifestations, which taught us an important lesson. Children with osteomyelitis caused by MAC (10, 11) or *Mycobacterium fortuitum* (12) mimicking LCH have been reported. Therefore, in addition to clinical and radiological features, LCH diagnosis should be based on pathological findings [especially immunohistochemical examination (8)] and diagnostic approaches should be adjusted as soon as possible.

Another lesson was that we did not have a deep understanding of several laboratory tests relevant to mycobacteria, and ignored the possibility of NTM infections. The Mantoux test and IGRAs can be used to detect an *in vitro* immune response against MTB, but the results of the Mantoux test can be confounded by NTM and previous BCG vaccination. Accordingly, a positive skin reaction in the Mantoux test can occur in patients with active TB infection, TB exposure in the past (latent TB infection), BCG vaccination in the past (live attenuated mycobacterial strain), and infection with a NTM variety (13). IGRAs (including T-SPOT.TB and QFT-G) measure IFN-γ in whole blood following incubation with MTB antigen, early secretory antigenic target-6 (ESAT-6), culture filtrate protein-10 (CFP-10), and tuberculosis 7.7 (TB7.7), which are absent in BCG strains and commonly encountered NTM, except *M. kansasii, M. gastri, M. marinum, M. szulgai*, and *M. riyadhense* (14). Therefore, IGRAs have high sensitivity for discriminating between NTM and MTB. As a traditional test for MTB, AFB smear microscopy cannot distinguish MTB from NTM, and further assessments are needed in some cases (14). qPCR is used widely to identify MTB infection rapidly, and presents high diagnostic value with high sensitivity and specificity. It is used commonly to ascertain if DNA or a unique sequence of the MTB genome is present in a sample. Moreover, it can be used to differentiate MTB from NTM efficiently (15). Therefore, clinicians should be aware of suspected NTM infections if AFB for clinical specimens is positive but the qPCR for MTB is negative. Finally, clinicians should turn to new technology to track infectious disease. In recent years, NGS has been employed widely thanks to its low cost. The applications of NGS in clinical microbiological testing are manifold and include mNGS, which allows for an unbiased approach to pathogen detection and enables broad identification of known and unexpected pathogens, or even the discovery of new organisms (16).

IFN-γ is a key cytokine produced predominantly by T-helper-1 cells and natural killer cells. It has a critical role in the cell-mediated immune cascade in response to invasion by intracellular pathogens. IFN-γ signaling is initiated at the IFNGR, which consists of a tetramer of two *IFNGR1* chains in complex with two *IFNGR2* chains. *IFNGR1* is the ligand-binding chain, whereas *IFNGR2* is the signal-transducing chain (17). IFNGR1 defects are autosomal recessive (AR) (complete or partial) or autosomal dominant (AD). In complete-type patients, *IFNGR1* is not expressed, and IFN-g is not produced; in partial-type patients, *IFNGR1* is expressed, with weak IFN-γ production. Clinical manifestations in patients with a complete AR *IFNGR1* deficiency are more severe than those in AR-partial or AD patients (18). Hematopoietic stem cell transplantation (HSCT) is the only known curative treatment for patients with complete AR *IFNGR1* deficiency, for which *IFNGR1* is not expressed, and IFN-γ is not produced. Some patients recover well after HSCT (12, 19). However, a high prevalence of graft rejection (even for transplants from a relative with an identical human leukocyte antigen) has been observed due to a high serum level of IFN-γ (19, 20).

Usually, the diagnosis of defects in *IFNGR1* is aimed initially at determining if any form of MSMD is present. This is done by determining the IFN-γ response in whole blood or peripheral blood mononuclear cells (17). However, in our case, the plasma level of IFN-γ was not measured, which was a limitation. Human IFN-γ levels and genetic patterns are traits that define the outcome of mycobacterial infection (21). For treatment of disseminated NTM disease associated with MSMD, aggressive, long-term antimycobacterial therapy to treat the acute infection and to prevent later recrudescence is required (1). In addition to antimycobacterial drugs, IFN-γ therapy is considered efficacious for improving the prognosis of partial *IFNGR1* deficiency, and successful cases have been reported (19, 22). In some cases, mycobacterial disease is well controlled by prolonged antibiotic treatment without treatment with recombinant IFN-γ (23). In our report, good recovery was observed with only anti-mycobacteria therapy without IFN-γ as an adjuvant. Usually, specific antimycobacterial therapies should be dependent upon the species identified and antimicrobial susceptibility to macrolide antibiotics. However, in this case, the infection was well controlled, and his condition improved significantly (though susceptibility tests for *M. intracellulare* indicated azithromycin resistance).

## Conclusions

Considering the vast range of microbial pathogens and infection sites in patients with MSMD, the importance of early identification of pathogens in the timely prescription of specific treatments in PIDs patients should be emphasized. We wished to: (i) improve the awareness of detecting early and persistent infections in very young patients; (ii) state our experience of treatment of disseminated MAC disease associated with *IFNGR1* deficiency.

## Patient Perspective

The initial anti-infection treatment was not very efficacious, and intermittent fever persisted. After several surgical procedures, my lumps were growing gradually, and eventually they ruptured. I felt more pain. My health seemed to be improving when I started taking azithromycin, rifampicin, and ethambutol every day. The lumps throughout my body became smaller and less painful. I had good tolerance to these drugs and did not experience adverse effects, such as skin rash, abnormal liver function, or blistering. I tried to walk by myself after 4 months of oral treatment. After more than 1 year of anti-*M. intracellulare* treatment, partial regression of bone lesions was observed in the most recent CT image, and I can walk normally now.

## Data Availability Statement

The raw data supporting the conclusions of this article will be made available by the authors, without undue reservation.

## Ethics Statement

The studies involving human participants were reviewed and approved by the Institute of West China Second University Hospital, Sichuan University. Written informed consent to participate in this study was provided by the participants' legal guardian/next of kin. Written informed consent was obtained from the individual(s), and minor(s)' legal guardian/next of kin, for the publication of any potentially identifiable images or data included in this article.

## Author Contributions

JJ and YZ collected the clinical data, carried out the initial analyses, and drafted the initial manuscript. QG and CW reviewed and revised the manuscript. All authors contributed to manuscript revision and approved the final version of the manuscript.

## Funding

This work was supported by a grant from Pediatric Clinical Research Center Foundation of Sichuan Province, China (No. 2017-46-4), National Science and Technology Major Project of China (No. 2018ZX10103–001), and Sichuan Science and Technology Program (Grant Number 2020YFS0042).

## Conflict of Interest

The authors declare that the research was conducted in the absence of any commercial or financial relationships that could be construed as a potential conflict of interest.

## Publisher's Note

All claims expressed in this article are solely those of the authors and do not necessarily represent those of their affiliated organizations, or those of the publisher, the editors and the reviewers. Any product that may be evaluated in this article, or claim that may be made by its manufacturer, is not guaranteed or endorsed by the publisher.
