# Supplementary figures and images for: Case Report: Disseminated Mycobacterium intracellulare Infection With More Than 1-Year Follow-Up in a Young Boy With IFNGR1 Deficiency
Source: Front Pediatr. 2022 Feb 28;10:761265. doi: 10.3389/fped.2022.761265 (PMC8914208; doi:10.3389/fped.2022.761265)

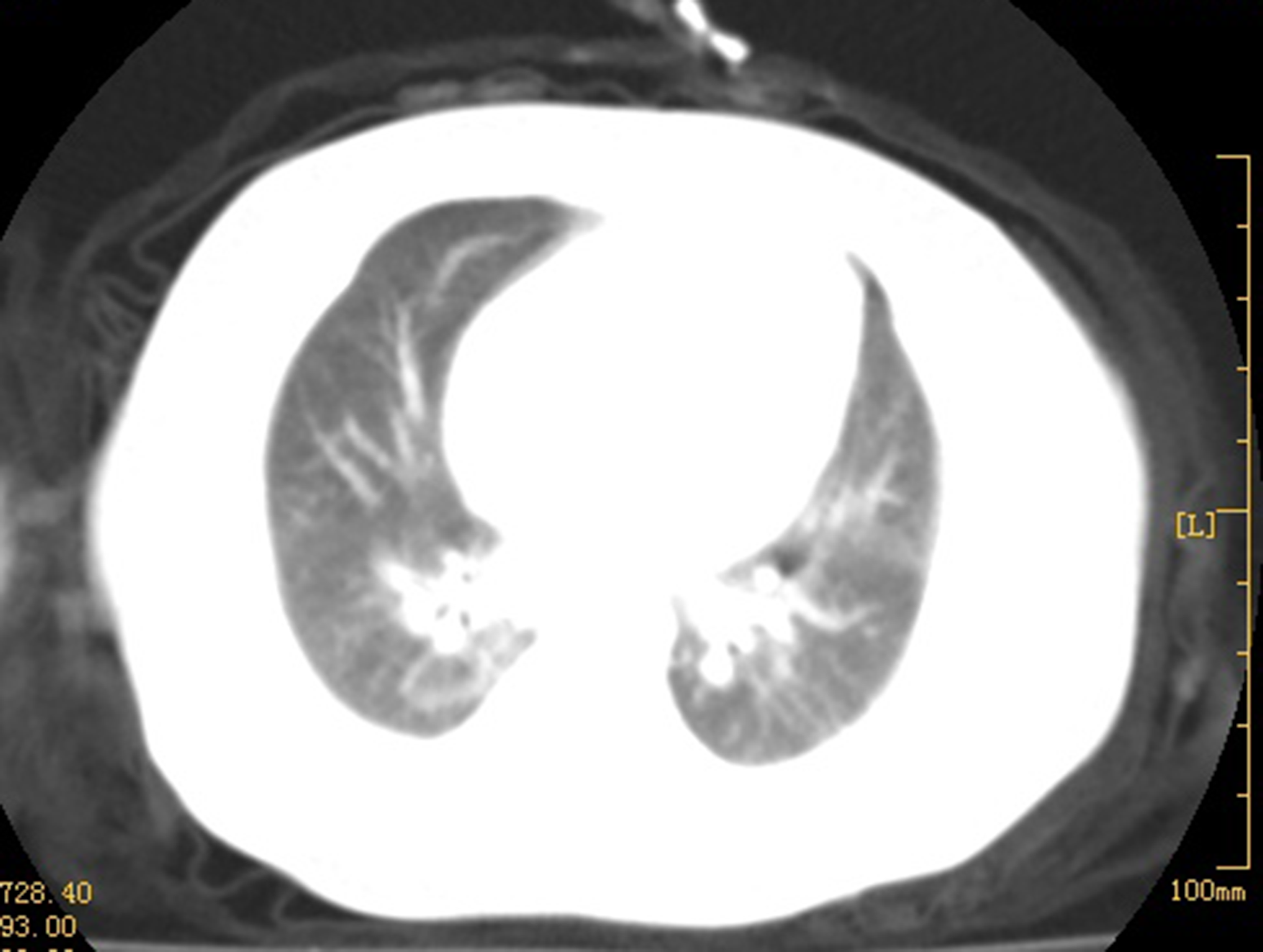

Supplement: Supplementary file 1 [file Image_1.TIF]

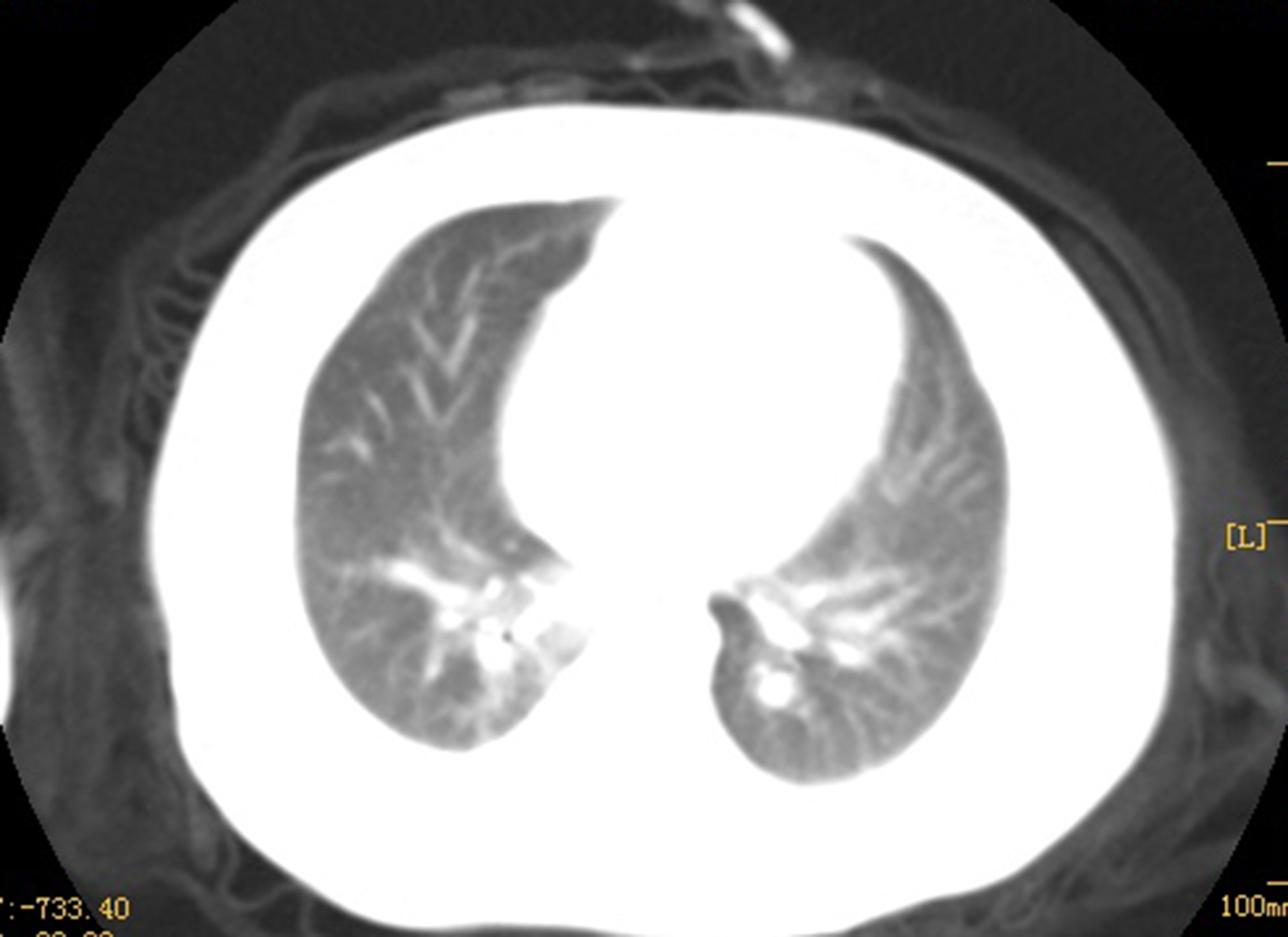

Supplement: Supplementary file 2 [file Image_2.TIF]

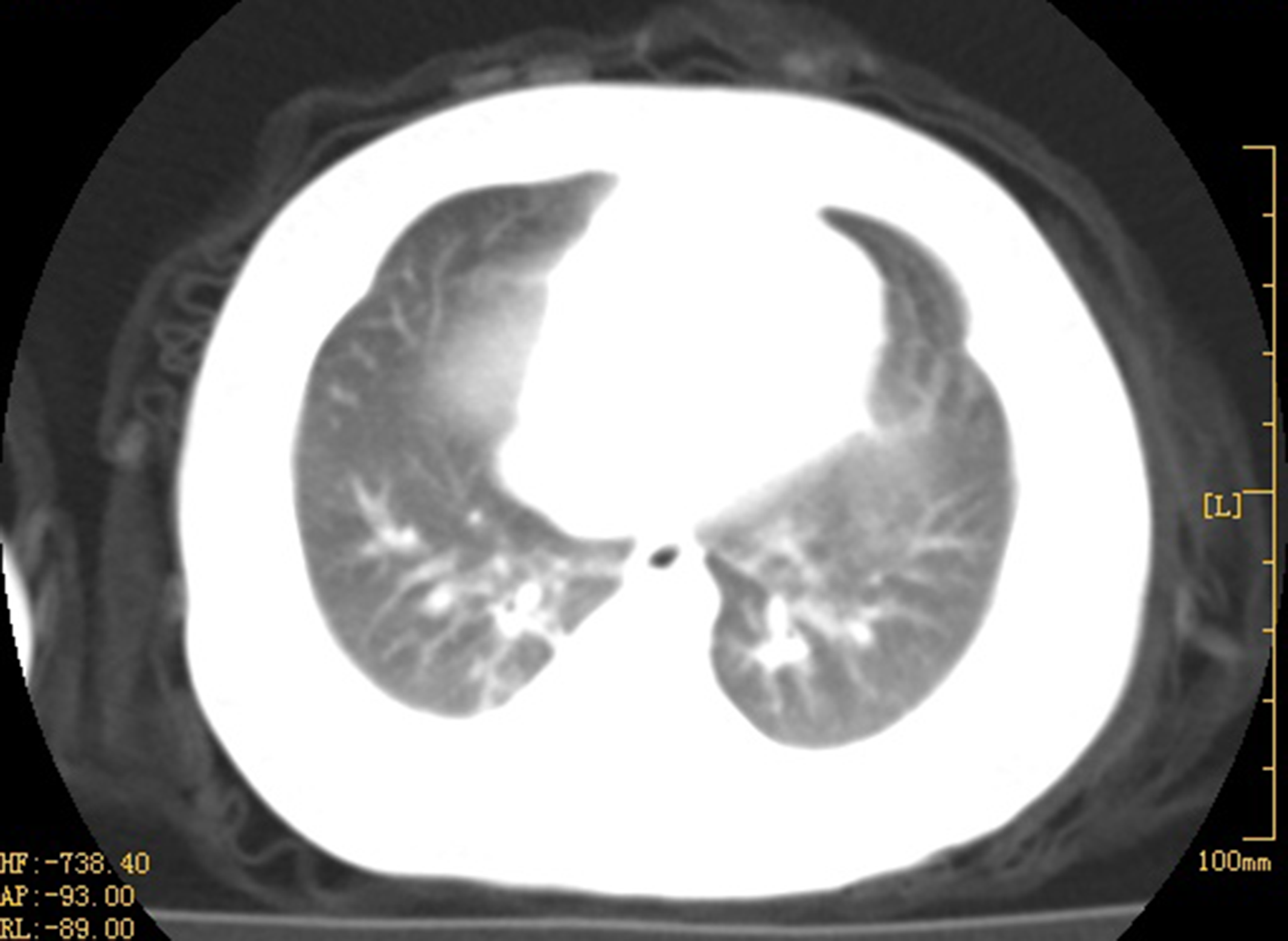

Supplement: Supplementary file 3 [file Image_3.TIF]

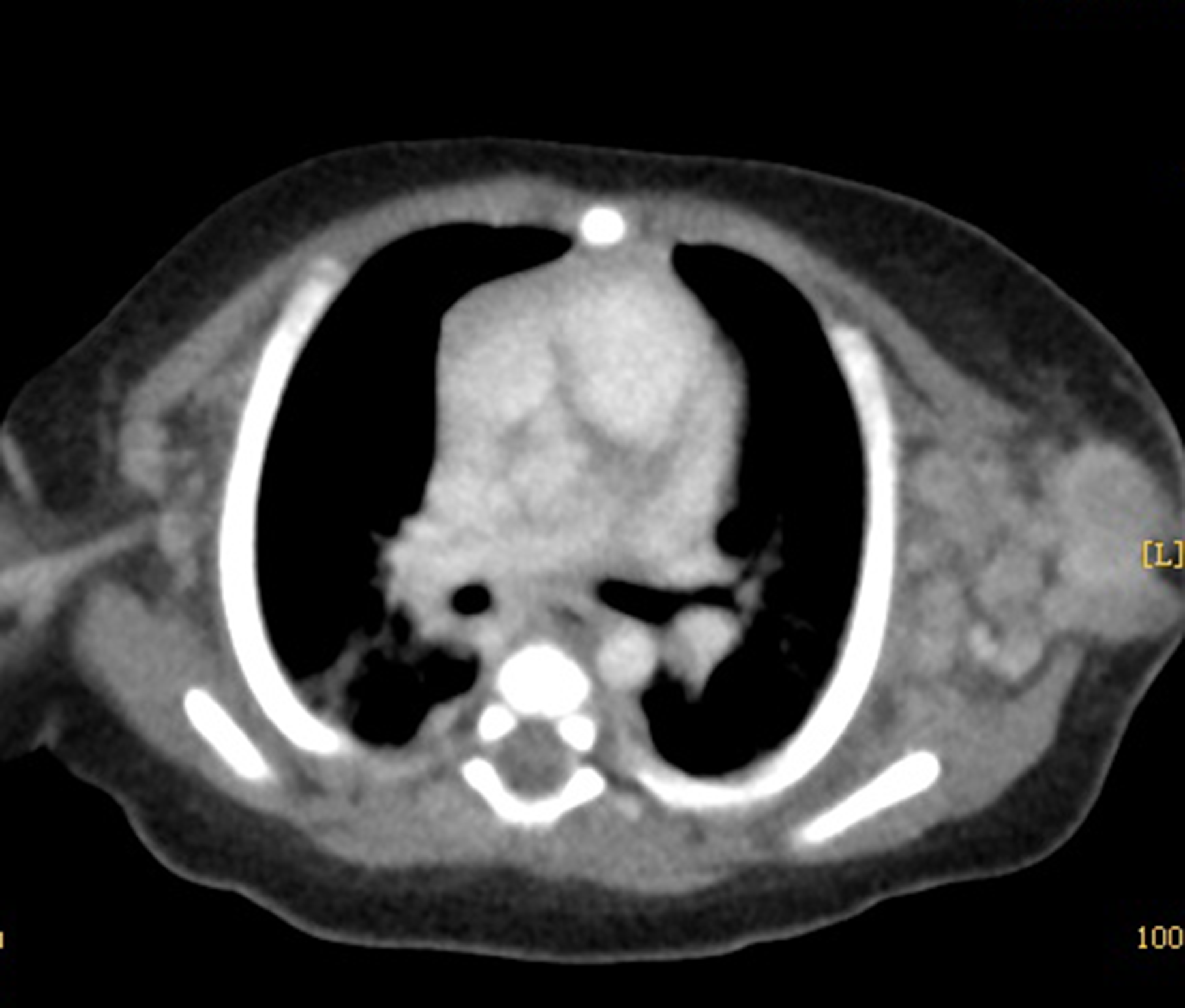

Supplement: Supplementary file 4 [file Image_4.TIF]

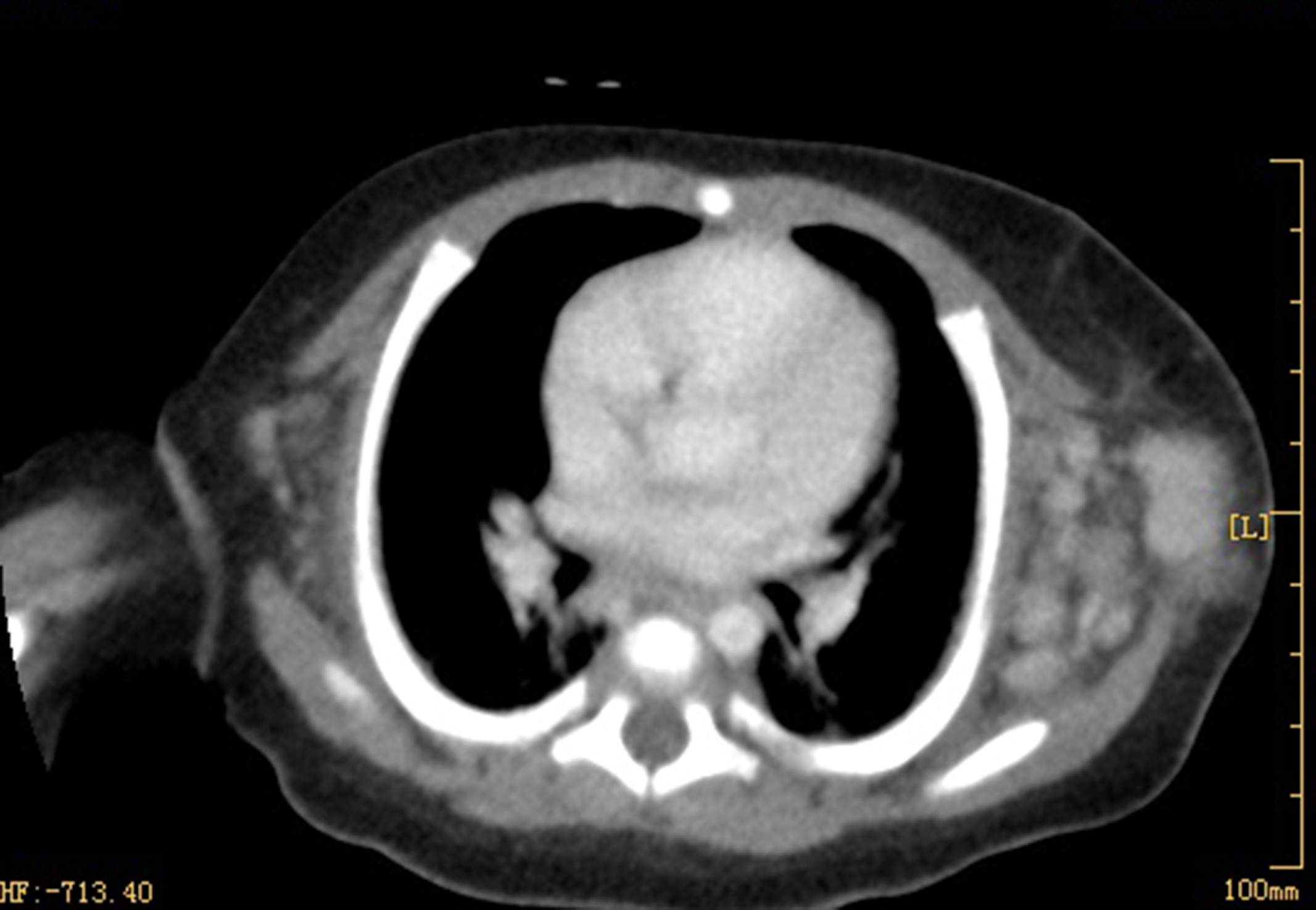

Supplement: Supplementary file 5 [file Image_5.TIF]

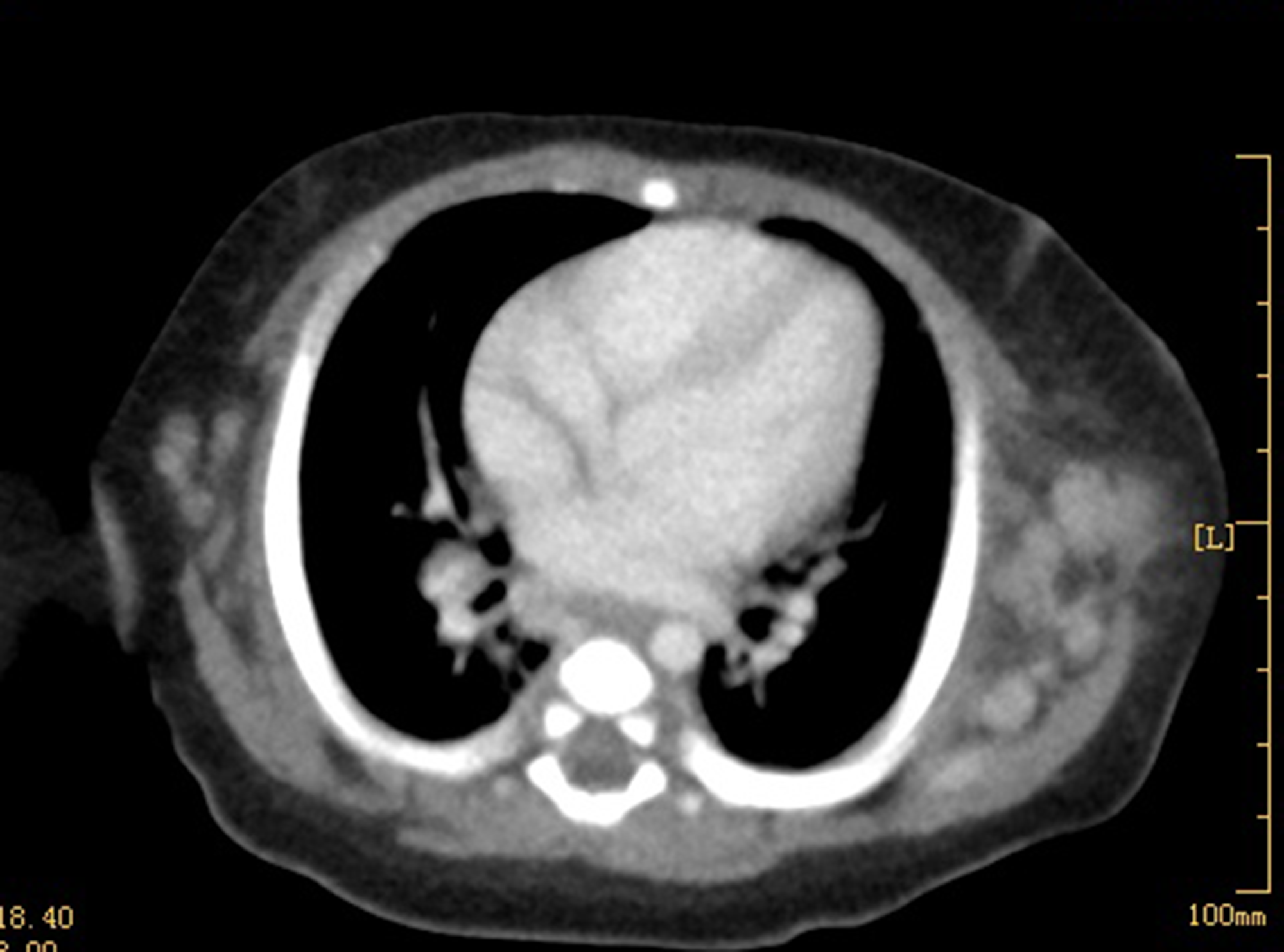

Supplement: Supplementary file 6 [file Image_6.TIF]

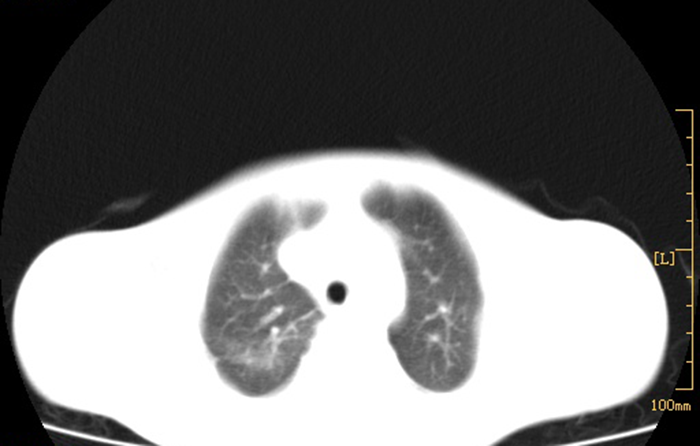

Supplement: Supplementary file 7 [file Image_7.TIF]

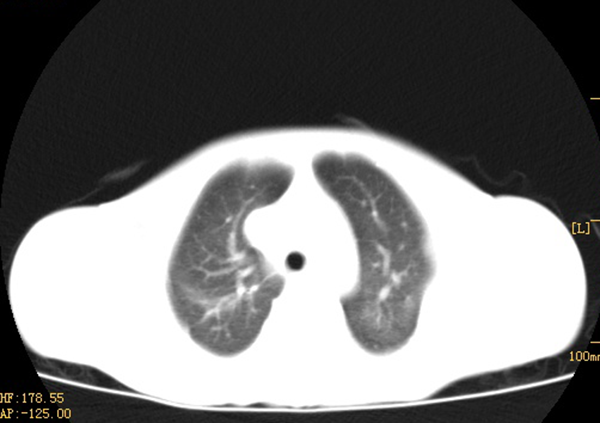

Supplement: Supplementary file 8 [file Image_8.TIF]

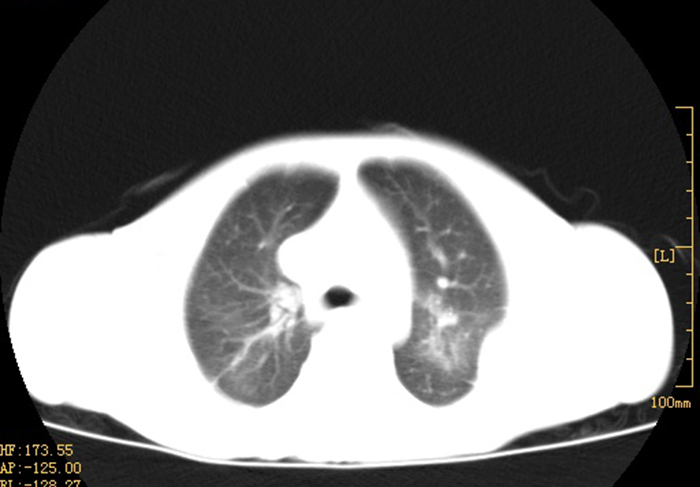

Supplement: Supplementary file 9 [file Image_9.TIF]
